# Modelling the genetic architecture of flowering time control in barley through nested association mapping

**DOI:** 10.1186/s12864-015-1459-7

**Published:** 2015-04-12

**Authors:** Andreas Maurer, Vera Draba, Yong Jiang, Florian Schnaithmann, Rajiv Sharma, Erika Schumann, Benjamin Kilian, Jochen Christoph Reif, Klaus Pillen

**Affiliations:** Institute of Agricultural and Nutritional Sciences, Martin Luther University Halle Wittenberg, Betty-Heimann-Str. 3, 06120 Halle, Germany; Interdisciplinary Center for Crop Plant Research (IZN), Betty-Heimann-Str. 3, 06120 Halle, Germany; Leibniz-Institute of Plant Genetics and Crop Plant Research (IPK), Corrensstr. 3, 06466 Stadt Seeland, OT Gatersleben Germany; Current address: University of Dundee at the James Hutton Institute, Invergowrie, Dundee, DD2 5DA UK; Current address: Bayer CropScience NV, Technologiepark 38, 9052 Ghent, Belgium

**Keywords:** Barley, Wild barley, Nested association mapping (NAM), Flowering time, Genome-wide association study (GWAS), Quantitative trait locus (QTL), Genomic prediction, Epistasis, Haplotypes

## Abstract

**Background:**

Barley, globally the fourth most important cereal, provides food and beverages for humans and feed for animal husbandry. Maximizing grain yield under varying climate conditions largely depends on the optimal timing of flowering. Therefore, regulation of flowering time is of extraordinary importance to meet future food and feed demands. We developed the first barley nested association mapping (NAM) population, HEB-25, by crossing 25 wild barleys with one elite barley cultivar, and used it to dissect the genetic architecture of flowering time.

**Results:**

Upon cultivation of 1,420 lines in multi-field trials and applying a genome-wide association study, eight major quantitative trait loci (QTL) were identified as main determinants to control flowering time in barley. These QTL accounted for 64% of the cross-validated proportion of explained genotypic variance (p_G_). The strongest single QTL effect corresponded to the known photoperiod response gene *Ppd-H1*. After sequencing the causative part of *Ppd-H1*, we differentiated twelve haplotypes in HEB-25, whereof the strongest exotic haplotype accelerated flowering time by 11 days compared to the elite barley haplotype. Applying a whole genome prediction model including main effects and epistatic interactions allowed predicting flowering time with an unmatched accuracy of 77% of cross-validated p_G_.

**Conclusions:**

The elaborated causal models represent a fundamental step to explain flowering time in barley. In addition, our study confirms that the exotic biodiversity present in HEB-25 is a valuable toolbox to dissect the genetic architecture of important agronomic traits and to replenish the elite barley breeding pool with favorable, trait-improving exotic alleles.

**Electronic supplementary material:**

The online version of this article (doi:10.1186/s12864-015-1459-7) contains supplementary material, which is available to authorized users.

## Background

Barley is among the oldest crop species human civilization was built on. Approximately 10,500 years ago, barley was domesticated in the Fertile Crescent [1,2], presumably followed by additional independent domestication events in East Asia [3,4]. Domestication and subsequent genetic selection led to gene erosion in most crop species’ gene pools [5,6], threatening future breeding advances. Utilizing the untapped biodiversity, present in wild progenitors is one promising approach to replenish the elite breeding pool with new favorable alleles [6-13]. The enriched diversity may be pivotal to boost the rate of genetic improvement and to cope with the anticipated enhanced effects of biotic and abiotic stresses due to climate change.

In this regard, time of flowering is expected to play a major role in future crop improvement. It is a key trait for the successful completion of a plant’s life cycle and, therefore, it has a strong impact on grain yield [14]. Flowering time indicates the transition from vegetative to reproductive stage, which is mainly influenced by environmental cues like day length (photoperiod) and prolonged exposure to cold temperatures (vernalization). In barley, the day length determining light signal is transmitted from a circadian clock oscillator, with *Ppd-H1,* a *PSEUDO-RESPONSE REGULATOR 7 (PRR7)* gene*,* in its center [15]. Under long day condition, *Ppd-H1,* through mediation of *CONSTANS (CO)*, promotes the expression of the floral inducer *Vrn-H3,* a homolog of the *Arabidopsis thaliana FLOWERING LOCUS T (FT)* gene [16]. On the other hand, *Vrn-H2,* a zinc-finger *CONSTANS*, *CO*-like and *TOC1 (CCT)*-domain protein (*ZCCT1*) acts as a repressor of *Vrn-H3* [17]. *Vrn-H2*, in turn, is repressed by *Vrn-H1,* an *APETALA1* family *MADS*-box transcription factor [18], which is up-regulated during vernalization. Thus, after vernalization, the repression of *Vrn-H3* is abolished and flowering is induced. Based on its vernalization requirement, winter barley and spring barley can be distinguished. Spring barley lacks the vernalization requirement due to a deletion of *Vrn-H2* [19].

Besides photoperiod and vernalization, there are also genetic mechanisms acting independently of environmental cues, so-called earliness *per se* [20]. Although several key regulatory cereal genes of flowering time were characterized and finally cloned during the last two decades, still little is known about the genetic architecture underlying flowering time regulation in temperate cereals, as compared to the model species *A. thaliana* [14,21-23]. So far, a holistic explanation of flowering time in a segregating germplasm population and the accurate prediction of a plant’s time of flowering, based on the combined action and interaction of major genes, is still not fully achieved in cereal species. Furthermore, it is reported that wild barley accessions possess a rich reservoir of beneficial alleles controlling flowering time [7,24,25].

**Figure 1:**
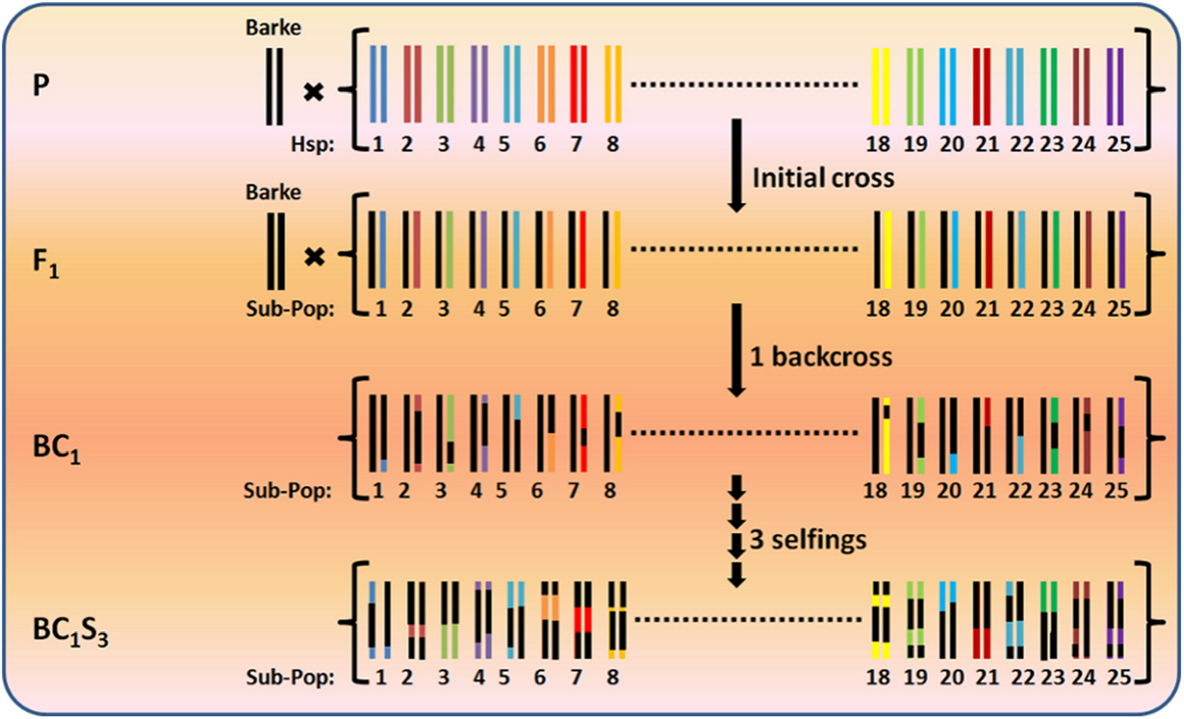
Development of the nested association mapping population HEB-25. HEB-25 is made of 25 families with 1,420 NAM lines in BC_1_S_3_. Per NAM line, one chromosome pair is illustrated as a double bar. Black and colored bars represent chromosome segments originating from Barke and the exotic donor accessions, respectively. At each SNP locus, HEB-25 is expected to segregate into 71.875% homozygous Barke, 6.25% heterozygous and 21.875% homozygous donor genotypes.

Nested association mapping (NAM) emerged as a multi-parental mapping design to investigate genomic regions with unprecedented genetic resolution and allelic variation by combining the advantages of linkage analysis and association mapping [26]. Hence, it facilitates the elucidation of a trait’s genetic architecture via genome-wide association study (GWAS). Until now, the NAM design was applied to the allogamous species maize and sorghum [26,27]. NAM populations for autogamous species like barley or wheat have not been developed, yet. In maize, the genetic dissection of various agronomic traits, including flowering time, has been investigated [28-34]. However, it was not possible to completely dissect the genetic architecture of flowering time in maize due to its complex inheritance and the multitude of involved small effect QTL. We developed the first NAM population in the autogamous species barley, termed ‘Halle Exotic Barley 25’ (HEB-25). The population results from initial crosses between the spring barley elite cultivar Barke (*Hordeum vulgare* ssp. *vulgare*, *Hv*) and 25 highly divergent exotic barley accessions, contributing an ideal instrument to study biodiversity. The exotic donors comprise 24 wild barley accessions of *H. vulgare* ssp. *spontaneum (Hsp),* the progenitor of domesticated barley, and one Tibetian *H. vulgare* ssp. *agriocrithon (Hag)* accession*.* Barke was selected since it was also used as a parent of a barley high-resolution mapping population [35] and as a genetic stock for mutation screening [36]. The exotic donors were selected from Badr *et al.* [37] to represent a substantial part of the genetic diversity that is present across the Fertile Crescent, where barley domestication occurred. To generate the nested population, F_1_ plants were backcrossed to Barke and, subsequently, selfed three times (Figure 1). In total, HEB-25 consists of 1,420 BC_1_S_3_ lines, divided into 25 HEB families of up to 75 lines per family (Additional file 1).

In the present study we investigated the genetic architecture of flowering time in barley. For this purpose, the NAM population HEB-25 was grown from 2011 to 2013 in multi-field trials to gather data on flowering time. By combining these data with high-density SNP marker information via genome-wide association studies and genomic prediction models, we could show that flowering time in barley mainly depends on a low number of large-effect QTL and epistatic interactions.

## Results and discussion

### Characterization of HEB-25

The inheritance of parental segments across the genomes of the 1,420 HEB lines was characterized through genotyping 5,709 informative, genic single nucleotide polymorphism (SNP) markers [35]. Marker saturation was high with an average genetic distance of 0.17 cM and a maximum gap of 11.1 cM between adjacent markers. Linkage disequilibrium (LD) among the 26 parents decayed rapidly (Additional file 2) enabling a high mapping resolution [26]. The SNP data revealed a low degree of genetic similarity between Barke and the donors, ranging from 40 to 54% (Additional file 1). Parents and the HEB-25 population could be clearly separated in a principal component analysis (PCA) (Additional file 3). Also, HEB families could be ordered in the PCA based on their geographical origin. These findings point to the high genetic diversity that is present among HEB-25 and its parents.

Diversity in HEB-25 was also visible phenotypically. During the seasons 2011 through 2013, HEB-25 was cultivated at the Halle University Experimental Field Station to collect flowering time data. The HEB lines flowered on average 68.1 days after sowing with a range from 51.0 to 98.9 days and a standard deviation of 6.5 days (Additional files 4 and 5). The broad variation in flowering time, covering almost 50 days among the 1,420 HEB lines, and a high heritability of 91.6%, as well as the genetic properties of the NAM population provided an excellent starting point to study the genetic architecture of flowering time through GWAS.

### Genome-wide association study

**Figure 2:**
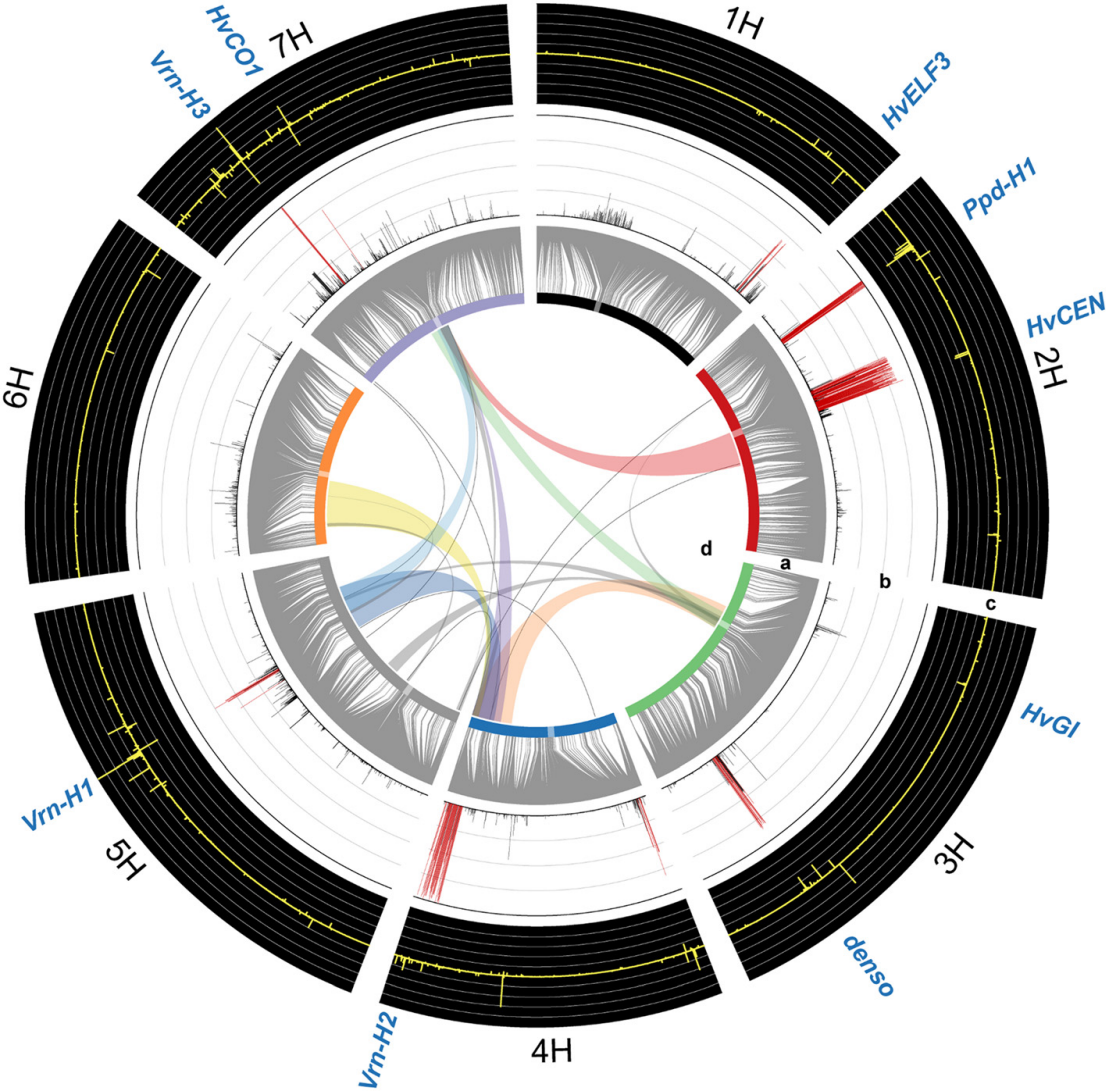
Genetic architecture of flowering time in HEB-25. Barley chromosomes are indicated as colored bars on the inner circle, centromeres are highlighted as transparent boxes. **a)** Grey connector lines represent the genetic position of SNPs on the chromosomes. **b)** Frequency of QTL detection in 100 cross-validation runs via GWAS (0 to 100, grid line spacing: 25); markers with > 50 detections are colored in red. **c)** Additive effect of the SNP obtained from the BayesCπ genomic prediction model. **d)** Links in the center of the circle represent significant (P_BON-HOLM_ < 0.05) di-genic interactions between SNP markers via GWAS. Clusters of significant SNP interactions are indicated by different colors. Position of candidate genes, potentially explaining major effects and epistatic effects, correspond to Table 1 and are indicated in blue outside the circle.

**Figure 3:**
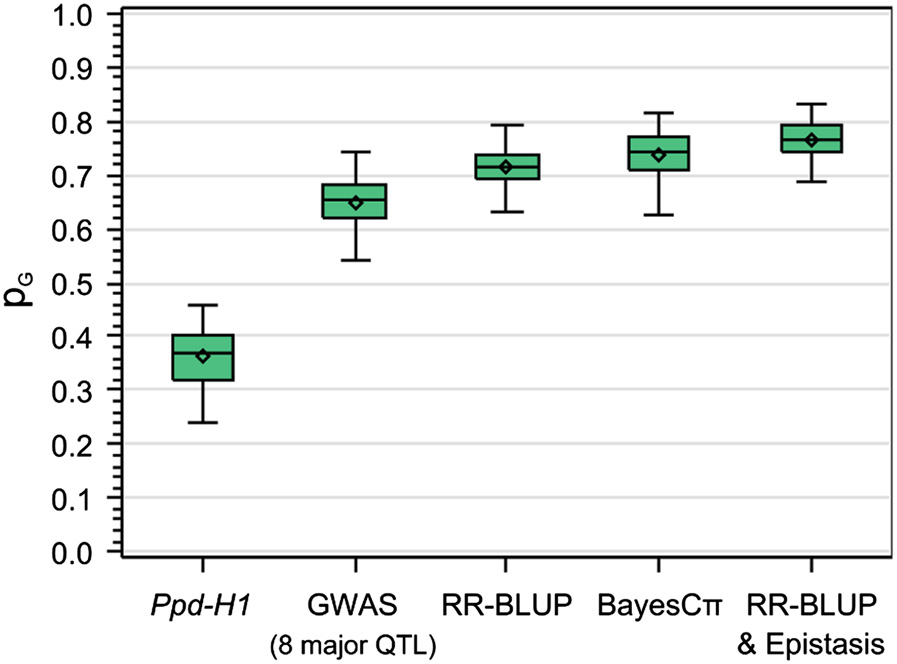
Cross-validated proportion of explained genotypic variance (p_G_) of different applied models. The box-whisker plots depict the variation of explained genotypic variance after 100 cross-validations. The tested QTL models are **(i)** the single SNP locus *Ppd-H1* (Mean p_G_ = 0.36), **(ii)** GWAS with peak markers, representing the eight major QTL indicated in Table 1 (Mean p_G_ = 0.64), **(iii)** the whole genome ridge regression best linear unbiased prediction (RR-BLUP, mean p_G_ = 0.71), **(iv)** the BayesCπ prediction (Mean p_G_ = 0.74), and **(v)** RR-BLUP including epistasis (Mean p_G_ = 0.77).

For GWAS, we initially applied the multiple linear regression Model-B with step-wise selection of cofactors, as outlined in Liu *et al.* [38]. Model-B was found most suitable to study traits across multiple related families [39], where a family effect and additional SNPs, selected as cofactors, are included in the model. GWAS revealed eight highly significant major QTL regions controlling flowering time with P_BON-HOLM_ < 1.0 E-10 (Table 1, Figure 2, Additional files 6 and 7). Testing the combined explanatory power of the single peak markers of the eight major QTL revealed a cross-validated explained proportion of genotypic variance (p_G_, [40]) of 64% (Figure 3). To check if genetic relatedness, as reported elsewhere [41], affects this parameter in HEB-25, we also estimated p_G_ for different sets of eight randomly chosen SNPs, excluding regions with significant QTL. However, since the cross-validated explained p_G_ remained low with an average of 8%, we conclude that genetic relatedness between individual lines does not play a major role in HEB-25. This emphasizes the power and precision of QTL detection in HEB-25, which may be a combined effect of the low extent of LD and the particular mating design, resulting in an elevated rate of chromosomal recombination. Thus, flowering time of barley can be reliably predicted based on eight major QTL. This finding is in contrast to flowering time regulation in the allogamous species maize and sorghum, where only small effect minor QTL were detected [28,42]. The most significant association in HEB-25 (P_BON-HOLM_ = 3.4 E-130) was observed on the short arm of chromosome 2H and explained a p_G_ of 36%. This SNP is directly located within *Ppd-H1*, the major determinant of photoperiod response in barley under long day condition [15]. Seven further genomic regions of extraordinary high significance were detected on chromosome arms 1HL, 2HS, 3HL, 4HS, 4HL, 5HL, and 7HS. All except one QTL (4HS) could be assigned to known flowering time genes (Table 1, Figure 2 and Additional file 7). Besides *Ppd-H1*, also the vernalization genes *Vrn-H1* and *Vrn-H2*, as well as the floral inducer *Vrn-H3* and its putative paralog *HvCEN* [43] exhibited highly significant effects. In addition, we could confirm the importance of gibberellic acid (GA) in flowering time regulation [44] through detection of *denso* [45] and *HvELF3* [46,47] as two further major QTL. Both genes are shown to be involved in GA biosynthesis [45,48]. So far, only the QTL on 4HS could not been referenced. This QTL, thus, remains a subject for further genetic investigations.

**Table 1:** **List of eight major QTL controlling flowering time in HEB-25**

**QTL**	**Chr** ^**a**^	**Pos** ^**b**^	**Range** ^**b**^	**Peak marker** ^**c**^	**No. Seg. Fam.** ^**d**^	**P**_**BON-HOLM**_^**e**^	**p**_**G**_^**f**^	**CV Freq.** ^**g**^	**Effect** ^**h**^	**CG** ^**i**^
QFt.HEB25-1b	1H	128.3	128.0-128.3	SCRI_RS_150786	25	2.41E-18	0.01	68	−1.4	*HvELF3* [46,47]
QFt.HEB25-2b	2H	23.0	16.8-23.8	BK_16	24	3.39E-130	0.36	100	−9.5	*Ppd-H1* [15]
QFt.HEB25-2c	2H	57.4	56.4-58.1	BOPA2_12_30265	25	2.25E-42	0.05	84	−3.0	*HvCEN* [35]
QFt.HEB25-3c	3H	108.4	107.8-109.2	BOPA1_ABC07496_ pHv1343_02	23	2.62E-62	0.04	83	−3.1	*denso* [45]
QFt.HEB25-4a	4H	3.5	3.5	BOPA2_12_31458	24	5.08E-15	0.05	82	3.2	
QFt.HEB25-4e	4H	113.4	113.4-114.3	SCRI_RS_216897	24	4.58E-17	0.02	100	2.2	*Vrn-H2* [17]
QFt.HEB25-5d	5H	125.5	125.5-125.8	BOPA1_4795_782	24	2.31E-33	0.06	60	3.8	*Vrn-H1* [18]
QFt.HEB25-7a	7H	34.3	25.9-34.3	BOPA2_12_30895	23	6.04E-69	0.07	100	4.1	*Vrn-H3* [16]

^a^Barley chromosome on which the QTL was determined.

^b^Genetic position of the peak marker and range of the QTL in cM, based on Comadran *et al.* [35].

^c^Marker of the QTL with the highest significance (peak marker).

^d^Number of families, in which peak marker is segregating.

^e^Significance of the peak marker, expressed as P_BON-HOLM_.

^f^Cross-validated proportion of explained genotypic variance of peak marker.

^g^Frequency of significant detections of the peak marker in 100 five-fold cross-validation runs.

^h^Difference between the wild genotype and the cultivated genotype in days until flowering. Early flowering effects of exotic alleles are indicated in red.

^i^Candidate gene, potentially explaining the QTL effect with reference.

The eight major QTL were located with high genetic precision, with four QTL restricted to confidence intervals of less than 0.9 cM (Table 1). In cases where gene-specific SNPs were available (i.e. *Ppd-H1* and *Vrn-H3*), exactly those SNPs revealed the highest significance within the respective QTL window (Additional file 6). The exotic alleles at *Ppd-H1* and *Vrn-H3* revealed the strongest effects, accelerating flowering time by 9.5 days and delaying flowering time by 4.1 days, respectively. The drastic effects of single QTL outline the high potential of introducing wild barley alleles from HEB-25 in order to adapt flowering time to environmental requirements and to enhance biodiversity in the elite barley breeding pool.

### *Ppd-H1* haplotype study

**Figure 4:**
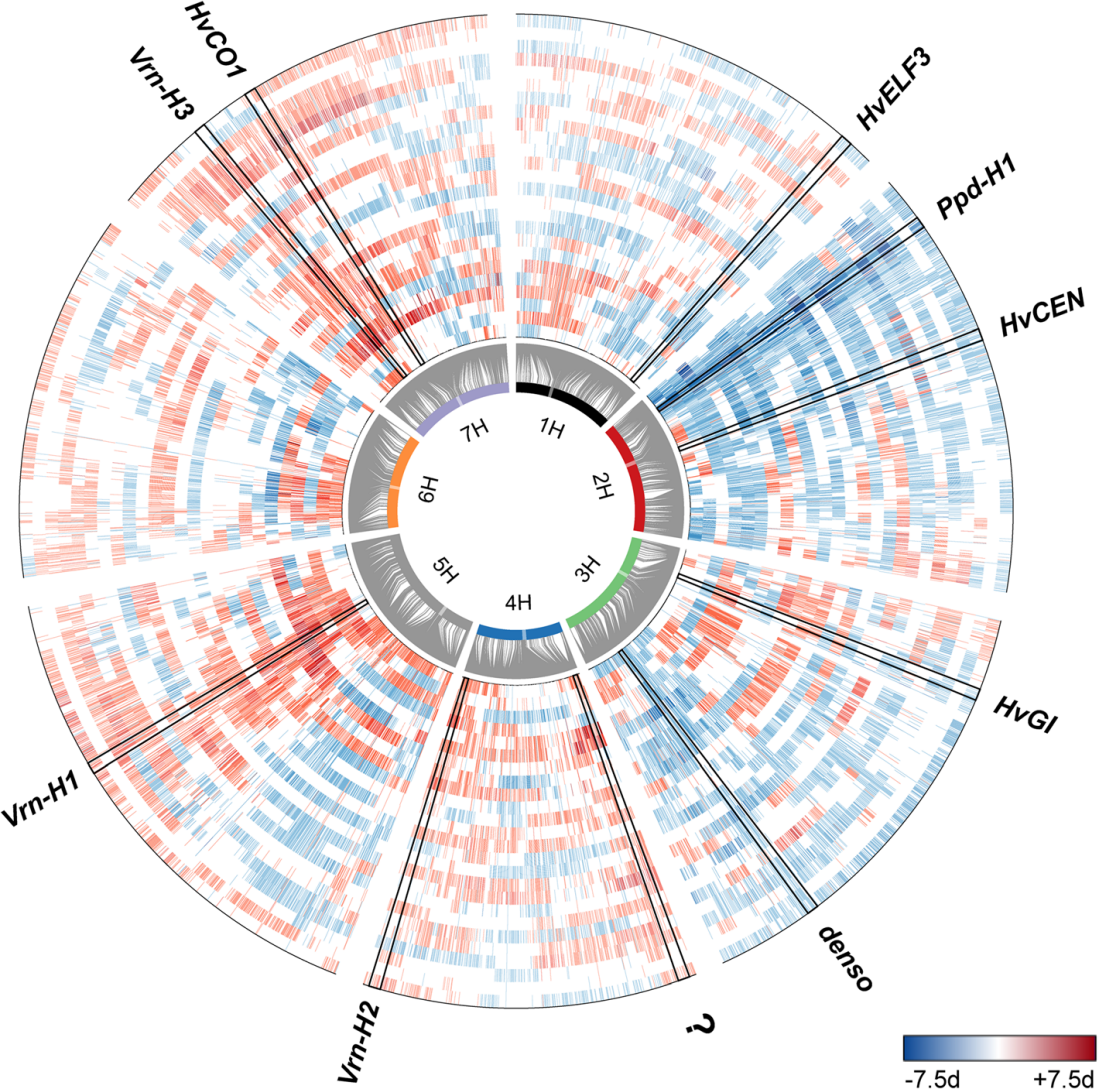
Visualization of family-wise SNP effects. Barley chromosomes are indicated as inner circle of colored bars, centromeres are highlighted as transparent boxes. Grey connector lines represent the genetic position of SNPs on chromosomes. Each track displays one HEB family (F01 – F25, from inside to outside). The heatmap indicates the difference in days between the donor and Barke genotype. Blue and red colors specify early and late flowering, respectively, caused by the donor genotype. White color indicates no SNP effect or SNPs monomorphic in the respective family. Candidate genes (Table 1) are indicated outside the circle. Black frames highlight their family-specific effects as indicated in Additional file 6.

**Figure 5:**
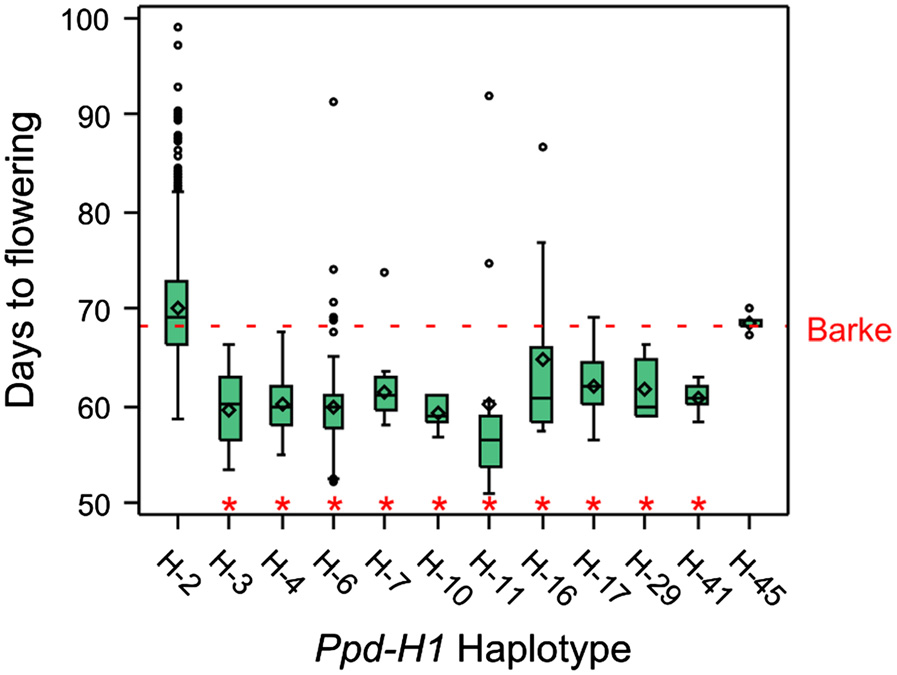
Box-whisker plots of flowering time BLUEs for *Ppd-H1* haplotypes. Green box-whisker-plots display the distribution of flowering time BLUEs of all HEB lines carrying the respective haplotype. Horizontal lines and diamonds indicate median and mean, respectively, for each haplotype. The extension of vertical lines indicates minimum and maximum observations, excluding outliers, which are indicated as circles. The red dotted horizontal line indicates the BLUE of cultivar Barke (68.2 days). H-2 represents the haplotype of the Barke genotype present in HEB lines. All haplotypes except H-45 differ significantly (P < 0.05) from H-2, as indicated by red asterisks. Further information to haplotypes is given in Additional files 1 and 8.

As we used bi-allelic SNP markers, additive effects were estimated across the NAM population. Theoretically, there may be up to 26 different alleles present at each locus in HEB-25. Thus, distinct alleles that show contrasting effects between families potentially escaped detection in our SNP-based GWAS. Contrasting effects are illustrated in Figure 4 and, in detail, in Additional file 6. For instance, SNPs at position 46.2 cM on chromosome 3H, which are tightly linked to *HvGI* [49], revealed opposing effects across HEB families. We tested the potential to integrate SNP haplotypes in the GWAS model for *Ppd-H1,* which exhibited the largest p_G_. After re-sequencing the last two exons and three introns of *Ppd-H1,* twelve haplotypes could be distinguished (Additional file 1). All *Hsp* donor haplotypes at *Ppd-H1* showed a significantly reduced flowering time (Additional file 8 and Figure 5), where a maximum reduction of flowering time was associated with H-6 (−11.1 days compared to elite barley haplotype H-2). Only the *Hag* haplotype H-45 did not differ from H-2. This finding confirms the presence of haplotype-specific effects present in HEB-25*.* Consequently, we expect the existence of further haplotype effects for other candidate genes controlling flowering time. The haplotype-based *Ppd-H1* model resulted in a slight increase of the cross-validated explained p_G_ from 36% to 38%. This finding implies that modelling haplotype-specific effects for a substantial portion of the barley gene space may result in an improved prediction of flowering time in HEB-25. However, a genome-wide re-sequencing of HEB-25 lines will be required to identify and distinguish those haplotypes.

### Applying genomic prediction models

To check whether we could further elucidate the genetic architecture of barley flowering time we applied genomic prediction models that considered all markers simultaneously. Genomic prediction evolved in animal breeding as a tool to predict a phenotype based on modelling a large set of SNP data [50]. It is used for selection of improved genotypes based on estimated genomic breeding values. Applying RR-BLUP [51] and BayesCπ [52] models, we could further increase the cross-validated explained p_G_ to 71% and 74%, respectively (Figure 3). These findings are in agreement with comparisons of multiple linear regression and genomic prediction of traits in bi-parental plant populations [53]. However, our p_G_ values substantially exceed the prediction accuracies of genomic prediction models reported in comparable studies [54-56], underlining the tremendous predictive power of HEB-25. Interestingly, compared to GWAS, only a few additional loci had non-zero effects in the BayesCπ model, indicating that flowering time is indeed mainly controlled by the eight major loci detected via GWAS.

We assume that important reasons for the slightly higher explained p_G_ of genomic prediction compared to GWAS are that minor QTL effects and marginally existing genetic relatedness [55,57] among HEB lines may be better modeled in the first case. Furthermore, modeling all makers simultaneously enables a better prediction of flowering time due to the estimation of family-specific QTL effects. This is indicated by the occurrence of opposing additive effects between HEB families alongside tightly linked SNPs (Figure 2 and Additional file 6).

### A model including epistasis to maximize the cross-validated explained p_G_

A final increase of the cross-validated explained p_G_ to an extraordinary high level of 77% was achieved by including di-genic epistatic interactions between significant main effect SNPs in the RR-BLUP model. This finding indicates that epistasis explains a portion of the ‘missing heritability’ [58] of flowering time regulation in barley, whereas in maize it does not [28]. The term ‘missing heritability’ is highly debated in quantitative genetics and refers to the observation that the explained genotypic variance of combined marker effects is usually lower than the heritability of the trait. Epistatic interactions between candidate genes may point to functional relationships and genetic networks [59]. Our findings indicate that the flowering time genes *HvGI*, *Vrn-H2, Vrn-H1* and *HvCO1* [60] on chromosomes 3H, 4H, 5H and 7H, respectively, are probably major players of di-genic epistatic interactions in HEB-25. All four genes potentially interact with each other as well as with further genes on additional chromosomes (Figure 2 and Additional file 9). These observations are in agreement with independent studies in barley and *A. thaliana* where these interacting genes were placed in a day length and temperature depending signaling network that controls flowering time [14,21-23]*.* It is, thus, likely that the observed interaction between the chromosomal regions in HEB-25 may be a function of the mentioned flowering time genes. As an example we refer to the potential interaction found between *Vrn-H1* and *Vrn-H2*. Epistatic interactions between these loci were already reported [17,61,62] and support the model that *Vrn-H2* is a long-day suppressor of flowering, that is itself suppressed by *Vrn-H1* following vernalization [63]. Barke is a spring type barley cultivar that lacks the vernalization requirement due to a deletion of *Vrn-H2* [19]. Hence, our findings may indicate that the epistatic interaction found between the two regions on chromosomes 4H and 5H is based on the presence (exotic allele) or absence (Barke allele) of *Vrn-H2*, the target of *Vrn-H1*. In general, the epistatic interactions detected in HEB-25 may provide hints for the presence of so far unknown functional networks of genes, which assist in fine-tuning flowering time in barley. Studies with knock out lines of these genes may be used to validate the observed interaction effects.

## Conclusions

The first barley NAM population HEB-25 provides great opportunities for future research and breeding. The genetic constitution of HEB-25 allows to carry out detailed studies on the genetic architecture of important agronomic traits, as exemplified by flowering time. The present study substantiated that flowering time in barley is primarily determined by large-effect QTL and epistatic interactions. This finding is in contrast to flowering time regulation in the allogamous species maize and sorghum, where only small effect minor QTL were detected [28,42], indicating that the mating system may control the genetic architecture of adaptive traits [28].

In future, the NAM population HEB-25 will be utilized in two directions: On the one hand, HEB-25 may support elucidating the genetic architecture of quantitatively inherited agronomic traits, ultimately resulting in cloning of yet unknown causal genes. On the other hand, HEB-25 will be exploited by breeders to enhance biodiversity of the elite barley gene pool. This may occur through introgression of favorable wild alleles with the aim to sustainably increase yield and stress tolerances against disadvantageous climate conditions like drought, heat and salt.

## Methods

### Development of the NAM population

The development of the NAM population ‘Halle Exotic Barley 25’ (HEB-25) was initiated in 2007 conducting crosses between the spring barley cultivar Barke (*Hordeum vulgare* ssp. *vulgare*) and 25 highly divergent exotic wild barley accessions. The latter were used as pollen donors. Twenty-four accessions, originating from Afghanistan, Iran, Iraq, Israel, Lebanon, Turkey, and Syria (*Hordeum vulgare* ssp. *spontaneum*), were selected to maximize the genetic diversity in HEB-25. One further accession, HID380, originating from Tibet, China, was classified as *Hordeum vulgare* ssp. *agriocrithon* (Åberg). F_1_ plants of the initial crosses were backcrossed with Barke as the female parent. Twenty BC_1_ plants per cross were subsequently selfed three times, using the single seed descent (SSD) technique to generate the next generations. The resulting BC_1_S_3_ generation consists of 1,420 individual lines, classified in 25 HEB families with 22 to 75 individual lines per family (Additional file 1). Subsequently, each HEB line was bulk propagated until BC_1_S_3:6_ to achieve sufficient seed numbers for field testing. No artificial selection was carried out during the development of HEB-25.

### Collecting single nucleotide polymorphism (SNP) data

SNP genotype data were collected at TraitGenetics, Gatersleben, Germany, for all 1,420 individual BC_1_S_3_ lines and their corresponding parents with the barley Infinium iSelect 9k chip consisting of 7,864 SNPs [35]. At each locus, three genotypes were differentiated, with an expected BC_1_S_3_ segregation ratio of 0.71875 : 0.0625 : 0.21875 for homozygous recipient (i.e. Barke), heterozygous and homozygous donor genotypes, respectively. In total, 1,027 monomorphic SNPs and 1,128 SNPs with high failure rates (i.e. no call in >10% of HEB lines) were excluded from the dataset, resulting in 5,709 informative SNPs for further analyses.

### Extraction of genomic DNA

DNA was extracted from leaf tissue of 1,420 single founder HEB plants in generation BC_1_S_3_. The subsequent seed propagation of HEB lines was based on these founder HEB plants. For Barke and the wild barley accessions leaf material from three to four plants was used to create bulked samples. The plants were cultivated in a glasshouse and 50 to 100 mg of leaf material was harvested for each sample. DNA was extracted according to the manufacturer’s protocol, using the BioSprint 96 DNA Plant Kit and a BioSprint work station (Qiagen, Hilden, Germany), and finally dissolved in distilled water at approximately 50 ng/μl.

### SNP mapping

The chromosomal positions of 3,391 out of 5,709 SNPs were taken from Comadran *et al.* [35]. The remaining SNPs were fitted next to the mapped SNPs applying chi-square tests of independence. Each non-mapped SNP was compared to each mapped SNP based on genotype segregation across all HEB lines. If two SNPs segregated completely independent from each other, i.e. in case of no linkage disequilibrium (LD), one expects to find all possible genotype combinations according to the product of their single locus genotype frequencies. However, in case of tight linkage, there should be a significant deviation from the expected genotype combination frequencies due to reduced recombination between these markers. Consequently, a high chi-square statistic and a low *P*-value likely indicate a tight linkage. Therefore, we assigned the position of the SNP with the lowest *P-*value (minimum: *P* < 0.001) to the non-mapped SNP under investigation. If there were more than one SNP with the same *P*-value, the position of the unmapped SNP was defined as the average of the minimum and the maximum position of the respective markers. In this way, all except six of the non-mapped SNPs were placed into the Comadran map.

### SNP calling

The differentiation of the HEB genotypes was based on an identity-by-state approach. Based on parental genotype information, the exotic allele could be specified in each segregating family. Thus, HEB lines that showed a homozygous exotic genotype were assigned a value of 2 and HEB lines that showed a homozygous Barke genotype were assigned a value of 0. Consequently, heterozygous HEB lines were assigned a value of 1. If a SNP was monomorphic in one HEB family but polymorphic in a second family, lines of the first HEB family were assigned a genotype value of 0 to keep a full genotype data set, which is a pre-requisite for the subsequent multiple regression analysis. For the same reason, missing genotypes were estimated applying the mean imputation (MNI) approach [64]. For this, each missing SNP value was replaced with the mean of the non-missing values of that SNP in the respective HEB family. Quantitative SNP genotypes were subsequently used for multiple regression analysis.

### Evaluation of genetic diversity

SAS 9.4 Software (SAS Institute Inc., Cary, NC, USA) was used to evaluate genetic diversity among parents and progenies of the HEB-25 population. Genetic similarities (GS) between HEB lines and their parents and among HEB lines were calculated with *Proc Distance*, based on a simple matching comparison between the three possible genotype states across all informative SNPs. In addition, we performed principal component analysis (PCA) using R [65]. First we applied PCA for the 26 parents (the cultivar Barke and 25 wild donors) based on the SNP matrix. The first two PCs explained 51.9 and 4.8% of the variation. Then, all progenies of HEB-25 were projected to the space spanned by the two PCs (Additional file 3) as outlined in detail elsewhere [66].

### Linkage disequilibrium (LD)

LD was calculated as r^2^ [67] between all mapped SNPs with the software package TASSEL [68]. For this purpose, heterozygous genotypes and SNPs with a minor allele frequency < 0.05 were excluded. LD was calculated across the 26 parents of HEB-25. LD decay across intra-chromosomal SNPs was displayed by plotting r^2^ between SNP pairs against their genetic distance. A second-degree smoothed loess curve [69] was fitted in SAS with *Proc Loess*. The population-specific baseline r^2^ was defined as the 95% percentile of the distribution of r^2^ for unlinked markers [70]. LD decay was defined as the distance, at which this baseline crossed the loess curve.

### *Ppd-H1* haplotype definition

For sequencing of the *Ppd-H1* locus on chromosome 2HS we used the following primers: PP05 (forward) 5′-GTGCAAAGCATAATATCAGTGTCC-3′ and PP04 (reverse) 5′-GGCCAAAGACACAAGAATCAG-3′. These primers amplify the last two exons and three introns of *Ppd-H1* covering the CCT domain that contains SNP22, the causal SNP of *Ppd-H1* [15]. Identical sequences were grouped into haplotypes. A detailed description of the sequencing is given in Jakob *et al.* [71].

### HEB-25 field trials

Between 2011 and 2013, three field trials were conducted at the ‘Kühnfeld Experimental Station’ of the University of Halle to gather phenotype data on flowering time. In 2011, the field trial was conducted with selfed progenies of BC_1_S_3_ lines (so-called BC_1_S_3:4_). Sowing occurred in single to five row plots with a length of 1.50 m and a distance of 0.20 m between rows. The number of rows per HEB line and the position inside the field trial depended on the number of available BC_1_S_3:4_ seeds. Lines with seed numbers lower than ten were planted in plots with a length of 0.50 m. In 2012 and 2013, the field trials were conducted with the selfed progenies in BC_1_S_3:5_ and BC_1_S_3:6_, respectively. Two replications per HEB line, arranged in two randomized complete blocks, were cultivated in 2012 and 2013. The plots consisted of two rows (30 seeds each) with a length of 1.50 m and a distance of 0.20 m between rows. All field trials were sown in spring between March and April with fertilization and pest management following local practice.

### Phenotypic data

The occurrence of flowering time was recorded as days after sowing, when the first awns were visible (BBCH49 [72]) for 50% of all plants of a plot. We performed a one-step phenotypic data analysis with SAS, using a linear mixed model with effects for genotype (i.e. 1,420 HEB lines), environment (i.e. 3 years) and interaction of genotype and environment. To estimate variance components, all effects were assumed to be random. Broad-sense heritability (h^2^) was estimated on an entry-mean basis. Best linear unbiased estimates (BLUEs) of flowering time were calculated for each genotype assuming fixed genotype effects.

### Genome-wide association study (GWAS)

For GWAS, we applied Model-B as outlined in detail by Liu *et al.* [38]. This model was found most suitable to carry out GWAS with multiple families [39]. It is based on multiple regression considering an SNP effect and a family effect in addition to cofactors, which control both population structure and genetic background [39]. Cofactor selection was carried out by applying *Proc Glmselect* in SAS and minimizing the Schwarz Bayesian Criterion [73]. The genome-wide scan for presence of marker-trait associations was implemented in the statistical software R [65], excluding cofactors linked closer than 1 cM to the SNP under investigation. The Bonferroni–Holm procedure [74] was used to adjust marker-trait associations for multiple testing. Significant marker main effects were accepted with P_BON-HOLM_ < 0.05. Additive effects for each SNP were estimated based on regression across but also within families. Significant marker trait associations were grouped to a singled QTL if the significant SNPs were linked by less than 5 cM and revealed additive effects of the same direction, i.e. both exotic alleles increased or decreased flowering time. In addition, a two-dimensional epistasis scan was carried out to identify pairwise marker interactions. For this, the GWAS Model-B was extended to cover a second main SNP effect and the interaction effect between the two SNPs.

### Haplotype-based association mapping for *Ppd-H1*

A haplotype-based association mapping test was implemented in HEB-25 to test for effects of haplotypes at *Ppd-H1*. We used the same GWAS procedure with cofactor selection as mentioned before. However, bi-allelic SNPs covering the region of *Ppd-H1* were replaced by a qualitative variable containing the defined *Ppd-H1* haplotype. BLUEs were determined for each haplotype. Subsequently, pairwise comparisons between all haplotype BLUEs were performed using the Tukey-Kramer [75] multiple comparison test.

### Genomic prediction

Based on BLUEs of the 1,420 HEB genotypes, two approaches for genomic prediction were applied considering additive effects: ridge regression best linear unbiased prediction (RR-BLUP [51]) and BayesCπ [52]. All statistical procedures for genomic prediction approaches were executed using R. We briefly describe the two models in the following.

Let *n* be the number of genotypes, *m* be the number of markers and *l* be the number of environments. The RR-BLUP model has the form *y* = 1_*n*_*μ* + *Xg* + *e*, where *y* is the vector of BLUEs of flowering time scores for all HEB genotypes across environments, 1_n_ denotes the vector of 1’s, μ is the overall mean, *g* is the vector of marker effects (for SNP markers, allele effects), *X* is the corresponding design matrix and *e* is the residual term. In the model we assumed that $g \sim N\left(0,{\sigma}_g^2\right)$, $e \sim N\left(0,\ {\sigma}_e^2\right)$, where ${\sigma}_g^2={\sigma}_G^2/m$ for SNP markers and ${\sigma}_e^2={\sigma}_R^2/l$. Here ${\sigma}_G^2$ and ${\sigma}_R^2$ are the genotypic and residual variance components obtained in the mixed model in the phenotypic data analysis. The penalty parameter is $\lambda =\left({\sigma}_R^2/l\right)/\left({\sigma}_G^2/m\right)$. The estimation of marker effects is then given by the mixed model equations [76].

The basic model of BayesC π is the same as RR-BLUP. However, all parameters are treated as random variables in a Bayesian framework. First, we defined the prior distributions as $g \sim N\left(0,{\sigma}_g^2\right),\kern1em e \sim N\left(0,\ {\sigma}_e^2\right)$. The prior of μ is a constant. The prior distribution of ${\sigma}_g^2$ is assumed to be zero with probability π and a scaled inverse chi-squared distribution with probability (1-π). The probability π is a random variable whose prior distribution is uniform on the interval [0,1]. The prior distribution of ${\sigma}_e^2$ is also scaled inverse chi-squared. A Gibbs sampler algorithm was then implemented to infer all the parameters in the model. It was run for 10,000 cycles and the first 1,000 cycles were discarded as burn in. The samples of *g* from all later cycles were averaged to obtain estimates of the marker effects.

### Cross-validation for additive models

The accuracy of the prediction of flowering time by GWAS and the two genomic prediction approaches were evaluated using five-fold cross-validations [77]. In each run of cross-validation, the estimation set included 80% of HEB lines, randomly selected per HEB family, while the remaining 20% of HEB lines were assigned to build the test set. For GWAS, we performed an association mapping scan within the estimation set and recorded the detected significant markers. To determine the cross-validated proportion of explained genotypic variance (p_G_), we estimated the effects of the significant peak markers within the estimation set and predicted the genotypic value of the lines in the test set [40]. We then calculated the cross-validated p_G_ as the squared Pearson product–moment correlation between predicted and observed genotypic values in the test set standardized with the heritability. The mean p_G_ in 100 cross-validation runs (20 times five-fold cross-validations) was taken as the final record. In addition, the number of significances for each SNP was cumulated across all runs and is referred to as QTL detection rate.

For genomic prediction we estimated the effects for all markers using the estimation set and predicted the genotypic value of the lines in the test set. The cross-validated p_G_ was calculated as in GWAS.

### Exploiting additive and additive times additive epistatic effects in genomic prediction

We extended the RR-BLUP based on main effects to model also epistasis for markers with significant main effects in the GWAS. The model is $y={1}_n\mu +{\displaystyle {\sum}_{i=1}^n{X}_i{g}_i}+{\displaystyle {\sum}_{j<l}\left({X}_j\cdot {X}_l\right)}{f}_{jl}+e$, where *y* is the vector of BLUEs of flowering time for all HEB genotypes, 1_n_ denotes the vector of 1’s, μ is the overall mean, *g*_*i*_ is the main additive effect of the i-th marker, *X*_*i*_ is the vector of marker indices, *f*_*jl*_ is the epistatic effects of the j- and the l-th marker, *X*_*j*_*X*_*l*_ is the point-wise product of the two vectors *X*_*j*_ and *X*_*l*_, and *e* is the vector of residual terms. Note that in the third term of the right hand side of the formula, the sum is not taken over all pairs of markers but only pairs of markers exhibiting a significant additive effect in the GWAS study performed previously. Hence, in different cross-validation runs, different pairs of markers were considered in the model. The model assumptions are similar to the usual RR-BLUP, except treating additive and epistatic effects separately. We assumed ${g}_i \sim N\left(0,{\sigma}_g^2\right)$, ${f}_{jl} \sim N\left(0,{\sigma}_f^2\right)$, where ${\sigma}_g^2={p}_G{\sigma}_G^2/m$, ${\sigma}_f^2=\left(1-{p}_G\right){\sigma}_G^2/p$. Here *P*_*G*_ is the cross-validated proportion of explained genotypic variance for genomic prediction, obtained previously by the RR-BLUP, only considering additive effects, m is the number of markers, p is the number of pairs of markers having significant additive effect. Therefore the penalty parameter λ is different for additive and epistatic effects.

Using the above extended model, for each cross-validation run we estimated the additive effects of all markers and epistatic effects of all pairs of markers exhibiting significant additive effects in GWAS using the estimation set. Then we predicted genotypic values of the lines in the test set and calculated the p_G_ in the same way as outlined above.

### Availability of supporting data

Raw data, including data on SNPs, *Ppd-H1* haplotypes and GWAS, and all other supporting data are provided as additional files.

## Additional files

Additional file 1:
**Genetic constitution of HEB-25: classification of families and donors.** Tabular overview of the genetic constitution of HEB-25, classifying the 25 families and donors and containing the *Ppd-H1* haplotypes. Additional file 2:
**LD decay of intra-chromosomal markers among HEB-25 parents.** Figure showing the LD decay of intra-chromosomal markers among HEB-25 parents by plotting r^2^ against the genetic marker distance. Additional file 3:
**Principal component analysis for HEB-25 and its parents.** Figure showing the relatedness of HEB-25 lines by plotting of the first two principal components of a principal component analysis for HEB-25 and its parents. Additional file 4:
**Distribution of flowering time.** Figure showing the frequency distribution of flowering time BLUEs across three field trials and illustrating contrasting phenotypes in the field. Additional file 5:
**Phenotype and genotype data for HEB-25.** Table listing the complete phenotype and genotype data of HEB-25 underlying this study as well as marker information. Additional file 6:
**Estimates of single marker GWAS and genomic prediction effects across HEB-25 and within individual HEB families.** Table listing the results of GWAS and genomic prediction across HEB-25 and within individual HEB families. Additional file 7:
**GWAS Manhattan plot for flowering time.** Figure displaying the GWAS results through plotting the significance and effects of markers in a Manhattan plot. Additional file 8:
***Ppd-H1*** **haplotype comparison.** Two tables contrasting the different *Ppd-H1* haplotype effects by comparison of their BLUEs. Additional file 9:
**Significant epistatic interactions via GWAS.** Table listing all significant (P_BON-HOLM_ < 0.05) epistatic interactions between SNPs that were obtained via GWAS.

## Abbreviations

BC_1_: Progeny of F_1_ after back-crossing with Barke
BC_1_S_3_: Progeny of BC_1_ after three rounds of selfing
BC_1_S_3:x_: Progeny of BC_1_S_3_ individual after x-3 rounds of bulk propagation
BLUE: Best linear unbiased estimate
cM: Centimorgan
F_1_: First generation after initial crosses
GA: Gibberellic acid
GS: Genetic similarity
GWAS: Genome-wide association study
*Hag*: *Hordeum agriocrithon*
HEB: Halle exotic barley
HID: *Hordeum* identity
*Hsp*: *Hordeum spontaneum*
*Hv*: *Hordeum vulgare*
LD: Linkage disequilibrium
MNI: Mean imputation
NAM: Nested association mapping
PC: Principal component
PCA: Principal component analysis
p_G_: Proportion of explained genotypic variance
QTL: Quantitative trait locus/loci
RR-BLUP: Ridge regression best linear unbiased prediction
SNP: Single nucleotide polymorphism
SSD: Single seed descent
