# Supplementary material for: Modelling the genetic architecture of flowering time control in barley through nested association mapping
Source: BMC Genomics. 2015 Apr 12;16(1):290. doi: 10.1186/s12864-015-1459-7 (PMC4426605; doi:10.1186/s12864-015-1459-7)
Supplement: Additional file 2: — LD decay of intra-chromosomal markers among HEB-25 parents. Figure showing the LD decay of intra-chromosomal markers among HEB-25 parents by plotting r2 against the genetic marker distance. [file 12864_2015_1459_MOESM2_ESM.pdf]

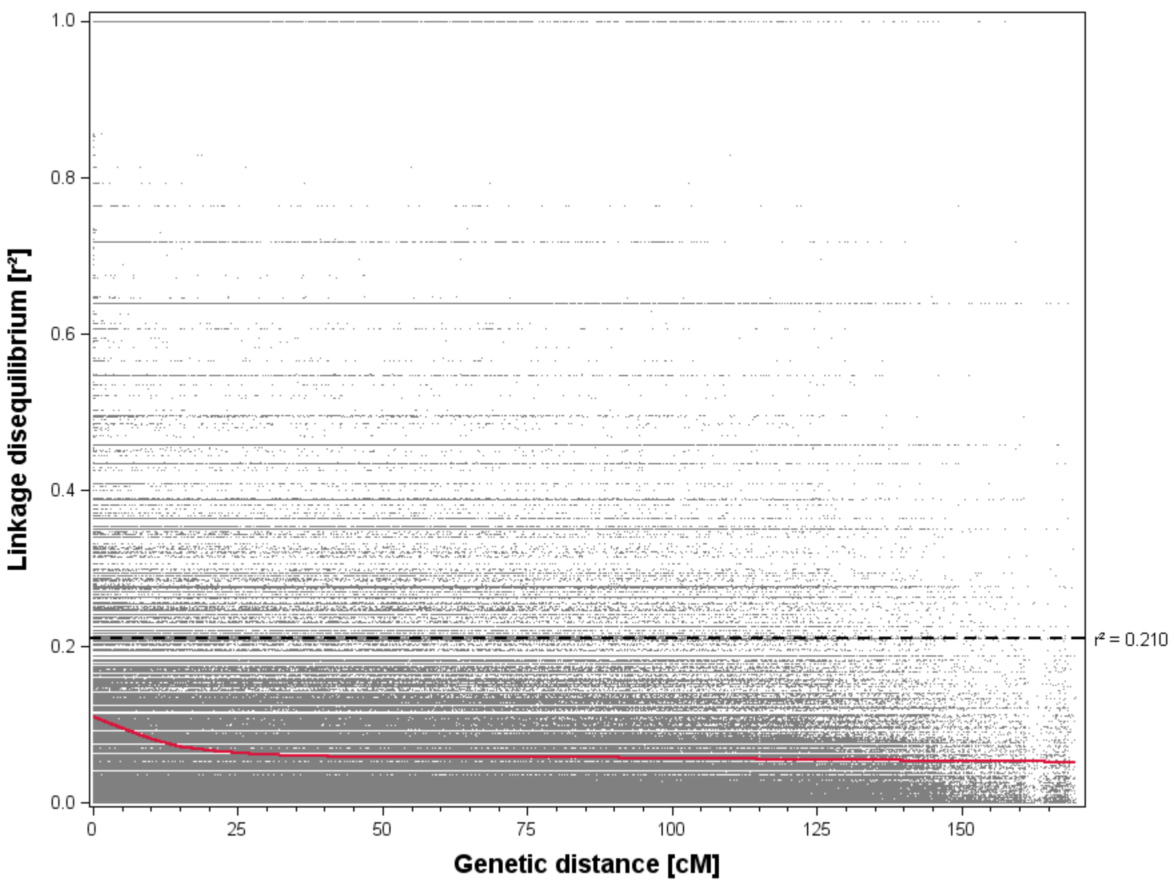

Additional file 2) LD decay of intra-chromosomal markers among HEB-25 parents. The red curve and the dashed black line indicate the loess fit and the threshold of LD, based on the 95th percentile of inter-chromosomal SNPs, respectively.
