# Supplementary material for: Modelling the genetic architecture of flowering time control in barley through nested association mapping
Source: BMC Genomics. 2015 Apr 12;16(1):290. doi: 10.1186/s12864-015-1459-7 (PMC4426605; doi:10.1186/s12864-015-1459-7)
Supplement: Additional file 3: — Principal component analysis for HEB-25 and its parents. Figure showing the relatedness of HEB-25 lines by plotting of the first two principal components of a principal component analysis for HEB-25 and its parents. [file 12864_2015_1459_MOESM3_ESM.pdf]

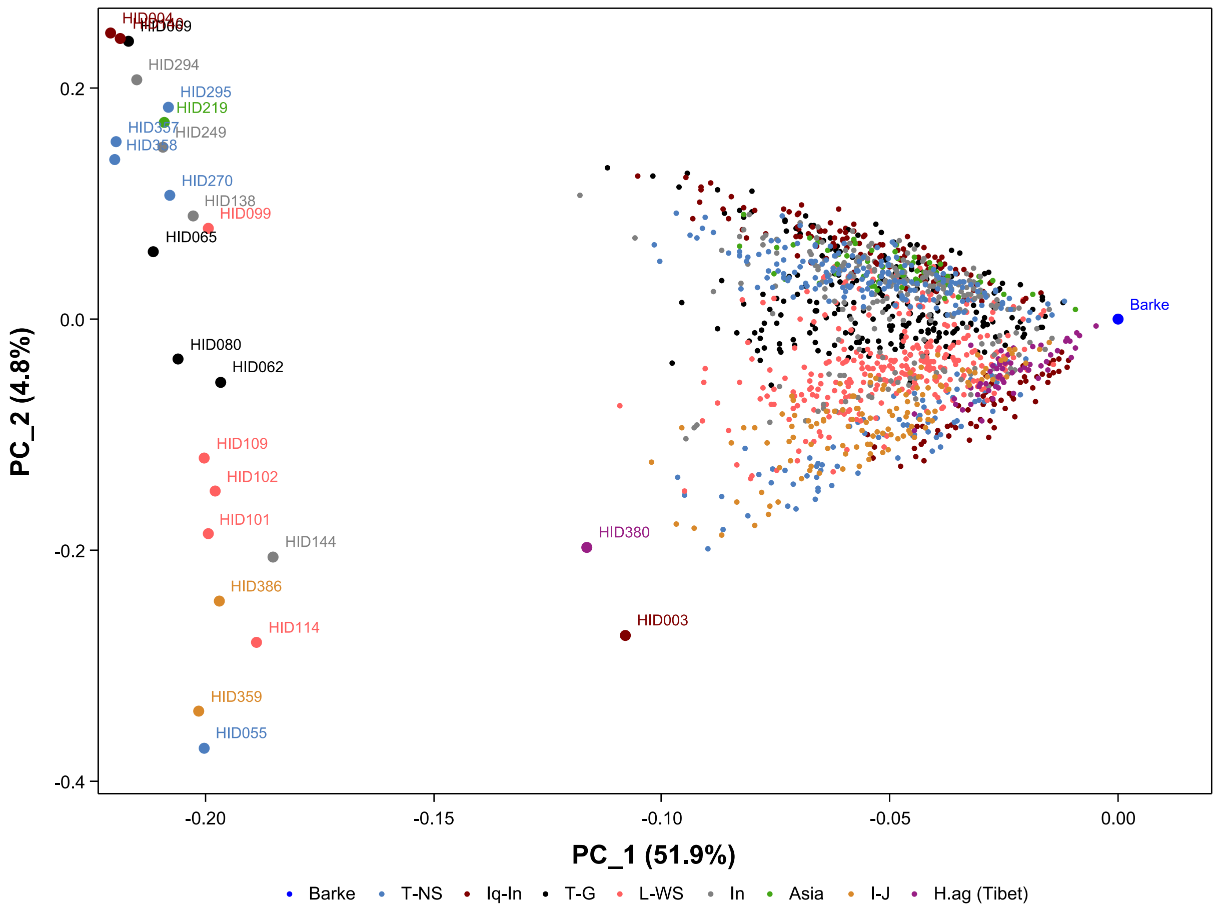

Additional file 3) Principal component analysis for HEB-25 and its parents. The two principal components (PCs) capturing the largest amount of variation are shown as x- and y-axis, respectively. Percentages in brackets denote the variance explained by the respective PC. Large circles indicate the 26 parents of HEB-25. Names of parents follow Table S1. Donors and HEB lines are color-coded based on the geographical origin of the donor accessions, given in Badr *et al.* [37]. Abbreviations: T-NS (Turkey near Diyarbakir and northern Syria), Iq-In (Northern Iraq and western Iraq), T-G (Turkey near Gaza), L-WS (Lebanon-western Syria), In (Southwestern Iran), Asia (Central Asia), I-J (Israel-Jordan), H.ag (Tibet).
