# Supplementary material for: Modelling the genetic architecture of flowering time control in barley through nested association mapping
Source: BMC Genomics. 2015 Apr 12;16(1):290. doi: 10.1186/s12864-015-1459-7 (PMC4426605; doi:10.1186/s12864-015-1459-7)
Supplement: Additional file 4: — Distribution of flowering time. Figure showing the frequency distribution of flowering time BLUEs across three field trials and illustrating contrasting phenotypes in the field. [file 12864_2015_1459_MOESM4_ESM.pdf]

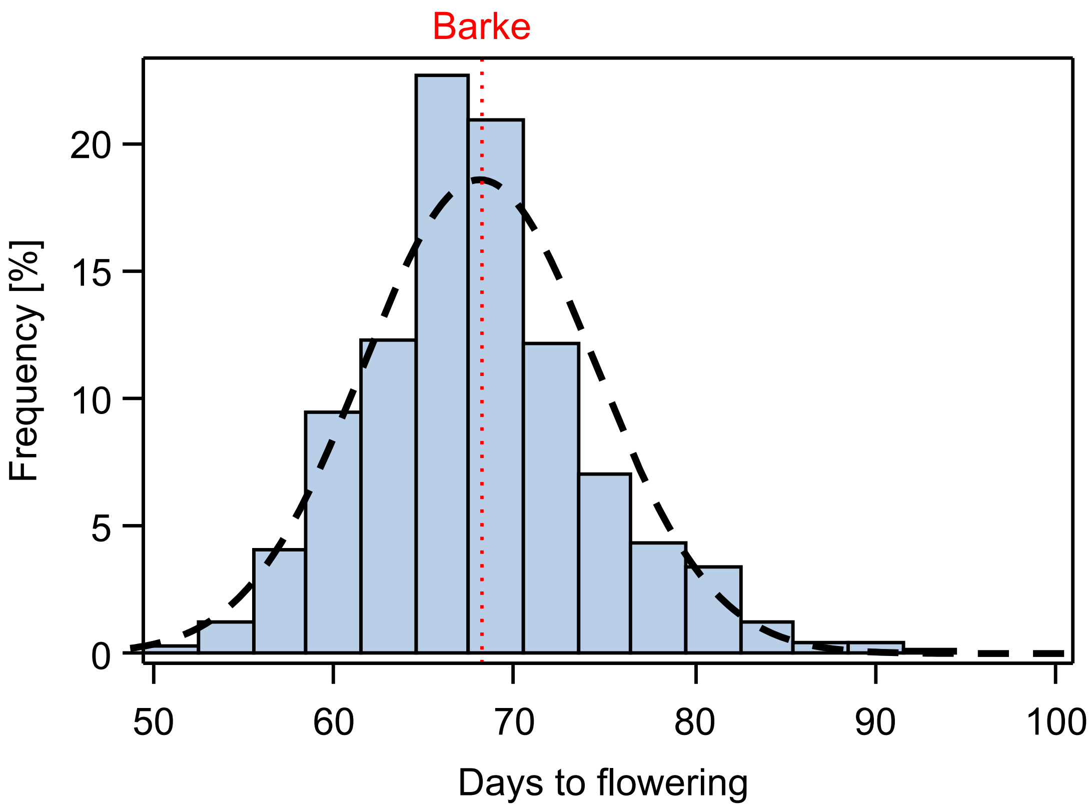

HEB-F06-113 | Barke | HEB-F12-130

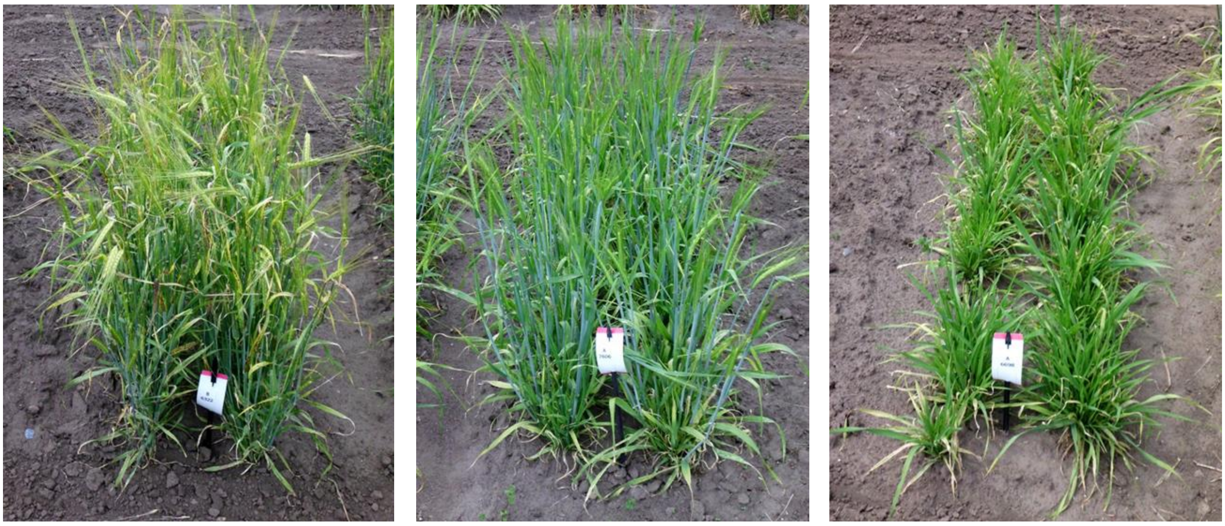

early | intermediate | late

Additional file 4) Distribution of flowering time. Upper part: Dashed line represents the normally distributed curve of flowering time BLUEs with $\mu = 68.1$ and $\sigma^2 = 41.7$. Barke flowered after 68.2 days and is indicated as red dotted line. **Lower part:** Phenotypes of two different HEB lines and Barke exhibiting contrasting speeds of flower development. All pictures were taken 83 days after sowing in 2012.
