# Supplementary material for: Modelling the genetic architecture of flowering time control in barley through nested association mapping
Source: BMC Genomics. 2015 Apr 12;16(1):290. doi: 10.1186/s12864-015-1459-7 (PMC4426605; doi:10.1186/s12864-015-1459-7)
Supplement: Additional file 7: — GWAS Manhattan plot for flowering time. Figure displaying the GWAS results through plotting the significance and effects of markers in a Manhattan plot. [file 12864_2015_1459_MOESM7_ESM.pdf]

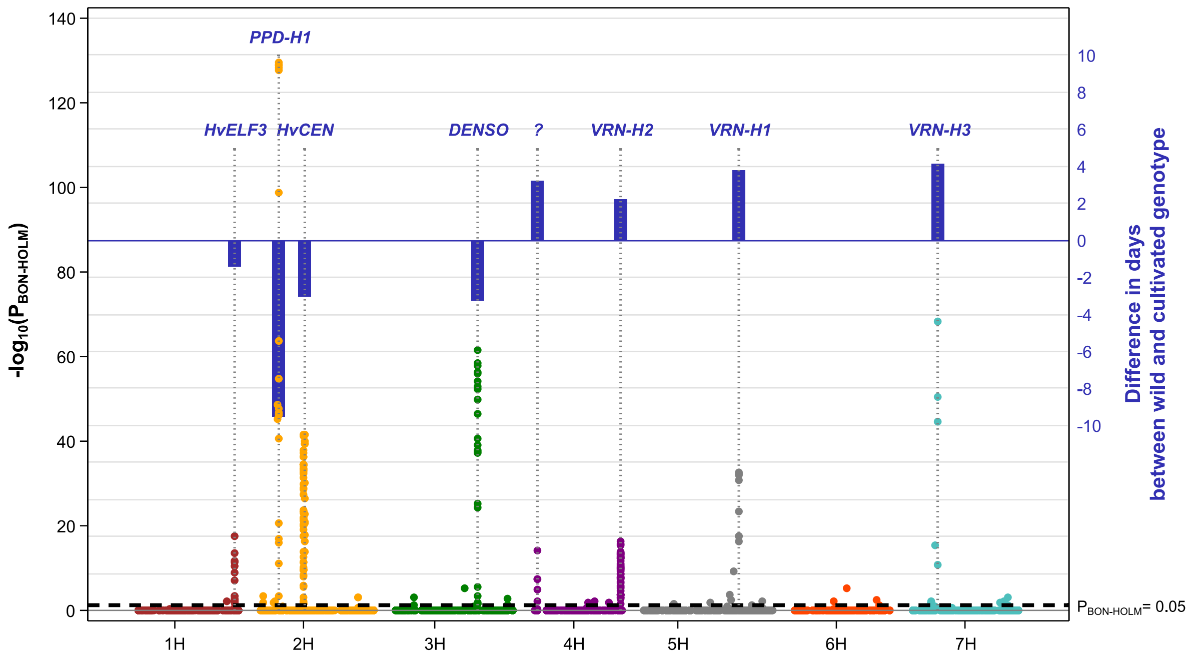

Additional file 7) GWAS Manhattan plot for flowering time. Colored dots represent the $P_{BON-HOLM}$ value of the marker-trait association, depending on the chromosome. The dashed black line indicates the significance threshold of 0.05. Ticks on the x axis indicate the position of the centromeres. Blue bars on top show the estimates for the difference between the wild genotype and the cultivated genotype in days. Candidate genes for the QTL are written above the peaks.
